# Progress in Radiotherapy

**Published:** 1903-02-21

**Authors:** 


### Progress in Radiotherapy.

(*Concluded from page* 339.)

Of 33 cases of inoperable cancer treated by $x$-rays Skinner[13] reports the disappearance of the growth in 2, permanent reduction of size in 14, and permanent arrest in 2; no effect in size being noticed in 14. There was general bodily improvement in 11; temporary improvement in 8; gain in body weight in 6; no influence in 27; hæmorrhage lessened in 8; not influenced in 1.

Out of the 33 there were 3 apparent cures, 13 permanently benefited and still improving, 2 were benfited, and in 3 treatment was discontinued too soon. Of the 3 cured cases 1 was a round-celled sarcoma of the neck of three years' standing. After the first treatment the pain disappeared. It was cured after the sixth application.

The second case was a recurrent carcinoma of the right breast. Sixty applications were given since March on the breast and deep lymphatics. The enlarged glands near the clavicle and in the pectoralis muscle had disappeared, and the patient had gained 10 lbs.

The third was one of a palpable tumour, probably sarcomatous, in the lumbar and upper sacral regions. There was great pain, but after 30 treatments the pain had disappeared and the tumour was palpable no longer. An operation was undertaken later for tubal pregnancy, and the surgeon could only find, of the original growth, three small modules the size of a pea.

Pratt[14] states that of 4 cases of epithelioma of the tongue treated he has succeeded in curing 2.

The use of the $x$-ray screen is warmly advocated by F. Gardiner[15] in the early diagnosis of phthisis pulmonalis. He states that the results are gratifying in early cases, as the rays will frequently reveal the condition before ordinary physical methods give any definite information. Three points are to be noticed:

(1) The loss of normal translucency shown in the lungs is due to infiltration and not simple congestion.

(2) Alterations in the shape of the chest are naturally more easily detected by the $x$-rays.

(3) Impairment of diaphragmatic movement is a very important sign, and one of the earliest to be noted. Early cases may show great restriction of movement, even though the loss of translucency is slight.

The apparatus used must be in perfect working order and without much flickering to give good results. A cavity if free from secretion stands out abnormally translucent, but if full may be invisible. Nodules may be seen in some cases. Collapse of the ribs is sometimes marked, and is a strong proof of diminished expansion. There is increased clearness in emphysema. The shadow cast by the phthisical lung is mottled; that shown in chronic pleurisy homogeneous. In acute pleurisy the diminished movement is easily seen, as is also effusion when present, the upper margin of which is easily demarcated.

Accidents in $x$-ray work have often been reported, but according to Cudman,[16] who has collected about 200 cases, their frequency is exaggerated. Of these 200 cases less than half were serious, and about a third occurred in $x$-ray workers themselves. More than two-thirds of the injuries occurred in the first two years of the treatment. He holds that the cause of $x$-ray injuries is unknown, but he believes it is due to a form of energy allied to the photographically active $x$-ray. The primary injury is to the nerves controlling the nutrition of the skin. There is good evidence of deep injury being produced without interference with the skin. The important factors contributing to $x$-ray burns are :—(*a*) Intensity of current ; (*b*) quality of tube ; (*c*) length of exposure ; (*d*) proximity of tube ; (*e*) idiosyncrasy of patient. In cases of injury the latent period before the development of symptoms ranged from a few minutes to three weeks. Five cases were longer than three weeks—two of these were five months. In a third of reported cases the appearance occurred within the first four days ; in half the cases before the ninth day.

Macintyre [17] reports a case of lupus of the nose, throat, and face of 46 years' duration which was cured by the high-frequency currents and the $x$ rays, and also gives two cases of malignant disease, one an epithelioma filling the whole nasal cavity and passing behind the upper lip to the gums near the roots of the incisor teeth, which entirely disappeared under treatment, and the second tumours of the breasts ; that on the left side had disappeared, while that on the right was making rapid progress towards recovery. He lays emphasis upon the great relief of pain and the disappearance of indurated masses, œdema, pressure symptoms, and the enlargement of glands even in cases considered surgically inoperable.

Further evidence of the use of the high-frequency currents in cases of hæmorrhoids comes from Pisani,[18] who has had success with a very severe case. Using four Leyden jars, from two to four ampères were applied for five to eight minutes at a time daily, by means of a properly constructed electrode, to the rectum. No pain was felt, and with the stronger currents only a sensation of warmth. The distressing spasmodic cortraction of the sphincter disappeared early, and the hæmorrhoids rapidly lessened in size and quickly and permanently disappeared at the end of 10 days' treatment. Constipation was not relieved, but the patient in five months was in excellent health.

Confirmation of the translucency of the body to intense light is supplied by Gottheid and Franklin,[19] who have performed a number of experiments in this direction. They directed a slightly convergent and strongly-condensed pencil of light from a large arc lamp taking 60 amps. upon various parts of the body. Negatives were prepared with sensitive films stuck to their back, and so covered that no light could reach them except through the negatives, and these were fixed to the back of the body of a strong mechanic aged 23. Light was then thrown upon the body in the direction of these plates.

At first they passed the light through the forearm (8 cms.), and after the 10 mins.' exposure the film showed the images of the negative and the forearm bones. Secondly, it was passed through the shoulder (16 cms.), and after 20 mins. the plate showed an even deposit, though less where the scapula cut off part of the light. Thirdly, the left abdominal region was tried (19 cms.) : 30 mins. was given, and the film showed an even and perfect exposure.

They thus attempted to pass the actinic vibrations through two layers of skin and varying thicknesses of subcutaneous fat, muscle, bone, and organs, the whole being permeated in every part with a circulating red fluid, to such an extent that it would affect a sensitized plate ; and their conclusions were, that light in proper concentration from a source of sufficient actinic power can be made to penetrate the entire thickness of the human body. Hence all the organs of the body are accessible to its influence. The distance of the organs being less than the whole thickness, the effect on them will be greater than on a plate at the back, and so the time required would not be so great.

Being efficacious in external maladies, it is reasonable to suppose that it might be of service to the internal organs.

[13] Med. Rec. Sept. 20. [14] Ibid. [15] Scotish Med. and Surg. Jour., Nov. [16] Amer. Jour. of Med. Sci., Aug. [17] Brit. Med. Jour., Oct. 25. [18] Treatment, June. [19] Med. Rec., April 19.

